# *In situ* K-edge X-ray absorption spectroscopy of the ligand environment of single-site Au/C catalysts during acetylene hydrochlorination†

**DOI:** 10.1039/d0sc02152k

**Published:** 2020-06-24

**Authors:** Grazia Malta, Simon A. Kondrat, Simon J. Freakley, David J. Morgan, Emma K. Gibson, Peter P. Wells, Matteo Aramini, Diego Gianolio, Paul B. J. Thompson, Peter Johnston, Graham J. Hutchings

**Affiliations:** Cardiff Catalysis Institute, School of Chemistry, Cardiff University Main Building, Park Place Cardiff CF10 3AT UK hutch@cardiff.ac.uk; Department of Chemistry, Loughborough University Loughborough Leicestershire LE11 3TU UK; Department of Chemistry, University of Bath Bath BA2 7AY UK; School of Chemistry, University of Glasgow Joseph Black Building Glasgow G12 8QQ UK; UK Catalysis Hub, Research Complex at Harwell, RAL Oxford OX11 0FA UK; School of Chemistry, University of Southampton Southampton SO17 1BJ UK; Diamond Light Source, Harwell Science and Innovation Campus Chilton Didcot OX11 0DE UK; XMaS, UK CRG, ESRF 71 Avenue des Martyrs 38043 Grenoble France; Department of Physics, University of Liverpool, Oliver Lodge Laboratory Liverpool L69 7ZE UK; Process Technologies, Johnson Matthey PLC Billingham TS23 1LB UK

## Abstract

The replacement of HgCl_2_/C with Au/C as a catalyst for acetylene hydrochlorination represents a significant reduction in the environmental impact of this industrial process. Under reaction conditions atomically dispersed cationic Au species are the catalytic active site, representing a large-scale application of heterogeneous single-site catalysts. While the metal nuclearity and oxidation state under operating conditions has been investigated in catalysts prepared from *aqua regia* and thiosulphate, limited studies have focused on the ligand environment surrounding the metal centre. We now report K-edge soft X-ray absorption spectroscopy of the Cl and S ligand species used to stabilise these isolated cationic Au centres in the harsh reaction conditions. We demonstrate the presence of three distinct Cl species in the materials; inorganic Cl^−^, Au–Cl, and C–Cl and how these species evolve during reaction. Direct evidence of Au–S interactions is confirmed in catalysts prepared using thiosulfate precursors which show high stability towards reduction to inactive metal nanoparticles. This stability was clear during gas switching experiments, where exposure to C_2_H_2_ alone did not dramatically alter the Au electronic structure and consequently did not deactivate the thiosulfate catalyst.

## Abstract 2

*In situ* chlorine and sulphur XAS shows a dynamic ligand environment around cationic Au single-sites during acetylene hydrochlorination.

## Graphical abstract

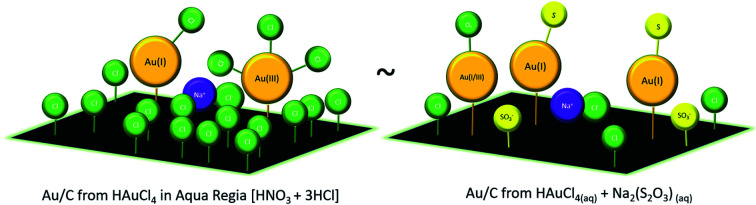

## Introduction

The commercialisation of carbon-supported gold catalysts (Au/C), as a replacement for toxic mercuric chloride (HgCl_2_/C), represents a significant reduction in environmental impact of large scale vinyl chloride monomer (VCM) production *via* acetylene hydrochlorination.^1–4^ Since the prediction that Au would be an effective catalyst,^5^ intensive studies to understand and optimise these catalysts have been on-going.^6–10^ These catalysts were originally developed using strongly acidic and oxidising solvents, including impregnation of HAuCl_4_ from *aqua regia* (Au/C-AR).^11,12^ The preparation and use of such Au/C catalysts at an industrial scale represents an economic and technical challenge, which hindered the validation of this type of catalyst. Moreover, the high activity of these Au/C materials is often coupled with poor lifetimes under extended testing. The reduction of the active cationic Au to metallic nanoparticles and the development of acetylene-oligomers on the catalyst surface at strong acid sites introduced from the *aqua regia* are the primary deactivation mechanisms.^1,13^ In comparison, catalyst preparation from aqueous HAuCl_4_ results in the formation of large Au nanoparticles which have limited activity.^14^ Alternative solvents to *aqua regia*, such as “organic *aqua regia* (OAR)”, have been developed and utilised for the preparation of mono and bimetallic catalysts;^15,16^ however, despite the appreciable catalytic performances this still does not provide a solution to large scale catalyst synthesis.

To obtain effective catalysts using aqueous metal-precursor solutions, strongly coordinating ligands are required to prevent nanoparticle formation.^17,18^ Johnston and collaborators,^1,19^ reported the use of soft donor ligands such as thiosulphate could produce a class of active and stable catalysts due to the increased stability constants of the Au–S species compared to the Au–Cl, avoiding the use of aggressive impregnation conditions. This catalyst (Au/C-S_2_O_3_) has been industrially validated as a replacement for the HgCl_2_/C. The synthesis is based on the *in situ* formation of a Au–thiosulfate complex before immobilisation onto carbon and the active form of the catalyst has been shown to consist of atomically dispersed cationic Au species under reaction conditions. We have previously demonstrated by *operando* X-ray Absorption Spectroscopy (XAS) that catalysts containing chloride or sulphur ligands had the same structure/activity correlation, with a Au(i) spectroscopic feature being proportional to VCM productivity and catalysts were shown to be comprise of atomically dispersed Au centres.^13^ The two catalysts (Au/C-AR and Au/C-S_2_O_3_) displayed differing induction periods; the Au/C-AR required 3 h to reach steady state activity, due to initial oxidation of Au(i) to Au(iii) and subsequent re-equilibration of metal oxidation states, while Au/C-S_2_O_3_ achieved steady state immediately due to higher stability of the Au(i) species with a similar Au(i) to Au(iii) ratio. In analogy with homogenous Au complexes,^20^ the choice of ligand and solvent, used during the catalyst preparation plays a major role in determining performance and stability.^21,22^

We now report a study using *in situ* K-edge S and Cl X-ray absorption spectroscopy, under relevant reaction conditions, to examine Cl and S speciation in the Au/C-AR and Au/C-S_2_O_3_ catalysts and correlate this to the observed catalytic activities. We show how the nature of the Cl and S evolve during the induction periods and at steady state to give information about the metal ligand environment and provide further information towards designing effective catalysts. The stability of the Au metal centre in the Au/C-S_2_O_3_ was studied in a *operando* Au L-edge XAS experiment, where reaction gases were switched to expose the catalyst to conditions that have already shown to deactivate the Au/C-AR catalyst.

## Results and discussion

**Fig. 1:**
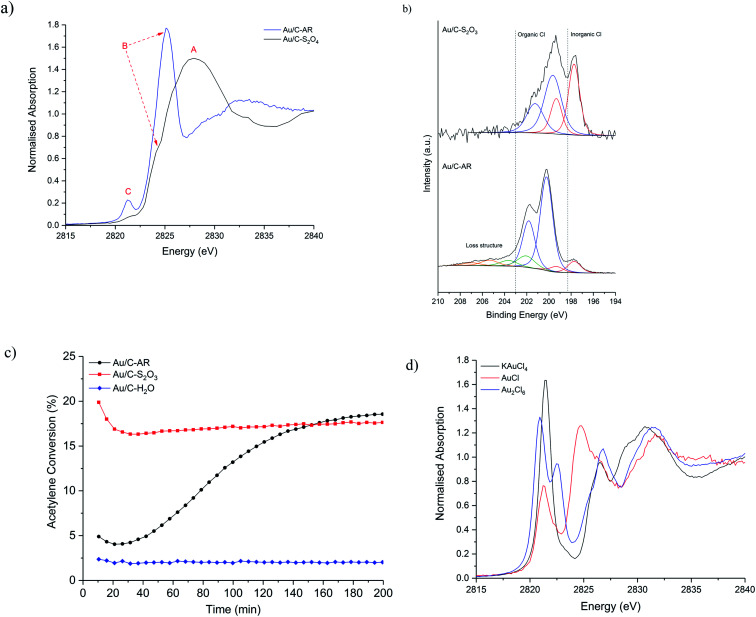
(a) Comparison of the normalised *ex situ* Cl K-edge XANES of the freshly prepared Au/C-AR (blue solid line) and Au/C-S_2_O_3_ (black solid line), (b) Cl(2p) core-level spectra for Au/C-AR and Au/C-S_2_O_3_ with associated binding energies; 197.7 eV – Au–Cl/NaCl (red), 200 and 202 eV – C–Cl functional groups (blue) and 203.4 and 205.2 eV loss structure associated with Cl species (orange). (c) Catalytic activity of the Au/C-AR (-●-, black), Au/C-S_2_O_3_ (-■-, red) and Au/C-H_2_O (-◆-, blue) (test conditions: 90 mg catalyst, 23.5 mL min^−1^ C_2_H_2_, 23.7 mL min^−1^ HCl and 2.7 mL min^−1^ Ar, 200 °C). (d) XANES of gold chloride standards. KAuCl_4_ (black solid line), Au_2_Cl_6_ (blue solid line) and AuCl (red solid line).

Initially, *ex situ* normalised Cl K-edge XANES spectra of fresh Au/C-AR and Au/C-S_2_O_3_ were compared (Fig. 1a). For comparative purposes a Au/C-H_2_O catalyst containing Au nanoparticles was also analysed (Fig. S1†). Three characteristic features, labelled as A, B and C, were observed in the XANES spectra of the catalysts with varying relative intensity. The absolute amount of Cl, determined from raw XANES spectra before data reduction^23^ differs in each sample (Fig. S2†); Au/C-AR has significantly more Cl (0.32) than Au/C-S_2_O_3_ (0.08) as expected from a preparation method using *aqua regia* compared to aqueous thiosulphate solution; the Au/C-H_2_O sample has lower amounts of Cl than either of the previous catalysts (0.03). These results are in accordance with Cl(2p) core-level XPS spectra (Fig. 1b and S3†), which verifies this difference in Cl concentration with Au/C-AR having 1.98 at% surface Cl and the Au/C-S_2_O_3_ 0.25 at% (Table S1†).

The XANES feature at 2828 eV, labelled (A), is assigned to the Cl 1s → 4p dipole allowed transition of an inorganic Cl^−^ species and it is comparable to the *ex situ* recorded reference material NaCl (Fig. S4†). The second feature at 2824–2826 eV, labelled (B), which is the predominant feature in Au/C-AR and a shoulder of the main edge for the Au/C-S_2_O_3_, can be assigned to a 1s → 3p* transition and associated with C–Cl functionality.^24^ This observation is in accordance with the assignments of XPS binding energies; Au–Cl/NaCl (197.7 eV), C–Cl groups (200 and 202 eV) and loss structure associated with high levels of carbon chlorination (203.4 and 205.2 eV)^25^ (Fig. 1b and S3†). Variation in the position of XANES C–Cl features is associated with the aliphatic/aromatic nature of the carbon species and the degree of chlorination;^26^ higher C–Cl energies, as seen in Au/C-AR, suggest a high degree of chlorination of the carbon surface. The high relative C–Cl content in Au/C-AR is rationalised as a result of chlorination from the highly acidic and oxidising *aqua regia* preparation. The XANES spectra of the Au/C-H_2_O catalyst is similar to Au/C-S_2_O_3_ however feature B (C–Cl) is more pronounced; aqueous impregnation of HAuCl_4_ on to the carbon support results is the deposition of metallic Au(0) nanoparticles and presumably consequent formation of a greater amount of C–Cl species.^10,13^

Bare activated carbon as received was also shown to contain both Cl^−^ and C–Cl species, but in negligible amounts compared to the catalysts; both XANES and XPS results suggest that after washing the carbon support with water (C-H_2_O) the inorganic Cl^−^ functionality was removed while treatment *aqua regia* (C-AR) was shown to be able to introduce C–Cl functionality (Fig. S5, S6 and Table S2†). The influence of water and *aqua regia* on the modification of the carbon surface, with and without the HAuCl_4_ metal precursor, has been also analysed *via* O 1s XPS. As expected, the *aqua regia* impregnation led to the highest oxygen content (Table S3†). However, the oxygen content does not seem to have direct influence on the catalysts' steady state activity when comparing the Au/C-AR and Au/C-S_2_O_3_ in our previous studies (catalytic data shown in Fig. 1c); this evidence also agrees with previous studies performed also on metal-free catalysts.^27^

The third feature in the Cl XANES at 2821 eV, labelled (C), is attributed to the Cl 1s → mixed Cl 3p and Au d orbital transition and is a pre-edge feature.^28^ The intensity of feature is dictated by the degree of covalency in the Au–Cl bond and is therefore sensitive to the electronic structure of the Au and its oxidation state. The hybridisation of Au 5d–6s orbitals, due to relativistic affects, allows the formally d^10^ electronic configuration of Au(i) to have molecular orbitals with Cl of sufficient character for electronic dipole allowed transitions. The feature is pronounced in Au/C-AR, while less intense in Au/C-S_2_O_3_ (Fig. 1a), showing that less Cl is associated with the Au and/or that the nature of the Au–Cl bonding is different, due to a change in bond covalency or lower oxidation state in the fresh Au/C-S_2_O_3_. Feature C is also negligible in the Au/C-H_2_O catalyst, in which the Au–Cl bond is almost absent (Fig. S1†).^13^ Given Au/C-S_2_O_3_ is prepared from a gold–thiosulfate complex made from HAuCl_4_, Au–Cl species could persist from the precursor. It is not possible to determine if the Au–Cl species is a discrete AuCl_*x*_ species or a partially chlorinated Au(S_2_O_3_)_*x*_ species, raising the question if multiple Au speciation exists. However, the clear difference in catalytic stability and induction behaviour of this catalyst shows that most Au centres in Au/C-S_2_O_3_ are significantly different from the Au–Cl_*x*_ in Au/C-AR (Fig. 1c). Similar considerations can be made in the comparison between the Au/C-AR and Au/C-H_2_O. From analysis of the Au–Cl pre-edge for the catalysts it is possible to determine if the dimeric Au_2_Cl_6_ species are present. The dimeric Au_2_Cl_6_ structure has two bonding Cl environments and Au–Cl bond lengths, *i.e.* terminal and bridged. This is reflected in the XANES spectrum of the dimeric standard (Au_2_Cl_6_) as a splitting of the pre-edge (Fig. 1d). On inspection and consistent with monomeric standard (KAuCl_4_), the XANES of both catalysts showed a single feature (Fig. 1d), indicating no significant population of Au dimers where present, supporting the observations made previously that the Au is present as mono-dispersed cationic species.^11^

**Fig. 2:**
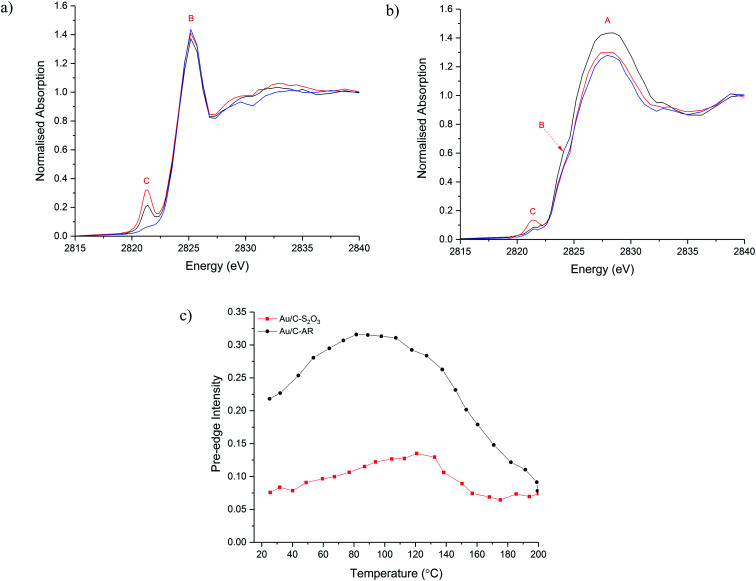
Cl K-edge XANES during the heating ramp of Au/C-AR (a) and Au/C-S_2_O_3_ (b) at start of heating ramp (black), maximum pre-edge intensity (red) and end of heating ramp (blue). (c) Pre-edge intensity against temperature for Au/C-AR (-●-, black) and Au/C-S_2_O_3_ (-■-, red).

To understand the evolution of Cl species during the acetylene hydrochlorination reaction, *in situ* Cl K-edge XANES characterisation of Au/C-AR and Au/C-S_2_O_3_ was performed. On heating to reaction temperature (200 °C) under He (Fig. 2a–c), the position of feature A, associated with inorganic Cl^−^ species, remains unchanged for both catalysts, with a slight decrease in normalised intensity in Au/C-S_2_O_3_. On the other hand, changes in feature C, associated with Au–Cl, were evident for both catalysts. In particular, the intensity of the pre-edge increased between 100–120 °C for the 2 catalysts, before reducing on further heating to 200 °C (Fig. 2c and S7†). This behaviour can be explained as following: on heating the catalysts the Au is oxidised to a higher oxidation state AuCl_*x*_, through migration of Cl from the carbon to the Au. This causes an increase in intensity of feature C as there is an increased Cl coordination number (CN) and a lower occupancy of the 5d–3p hybridized orbital. The small decrease in the C–Cl band in Au/C-S_2_O_3_ concurrent with the increase in feature C supports this hypothesis; the absence of any notable C–Cl population change in Au/C-AR is attributed to the significantly higher initial signal intensity, masking any small change in signal from Cl migration. The reduced intensity of feature C above 110–130 °C is due to the decomposition of Au(iii) Cl to Au(i) Cl, where fully occupied d orbitals limit the pre-edge feature,^29^ in agreement with Au L_3_-edge experiments.^13^ This observation suggests that the C–Cl species can be mobile and interact with the Au species at elevated temperatures by acting as a reservoir of Cl functionality. It is important to acknowledge that while changes between Cl environments can be inferred, the intensities of allowed 1s → 4d transitions cannot be proportionately compared with feature C, which is dependent on Cl 3p–Au 5d/6s hybridisation.

**Fig. 3:**
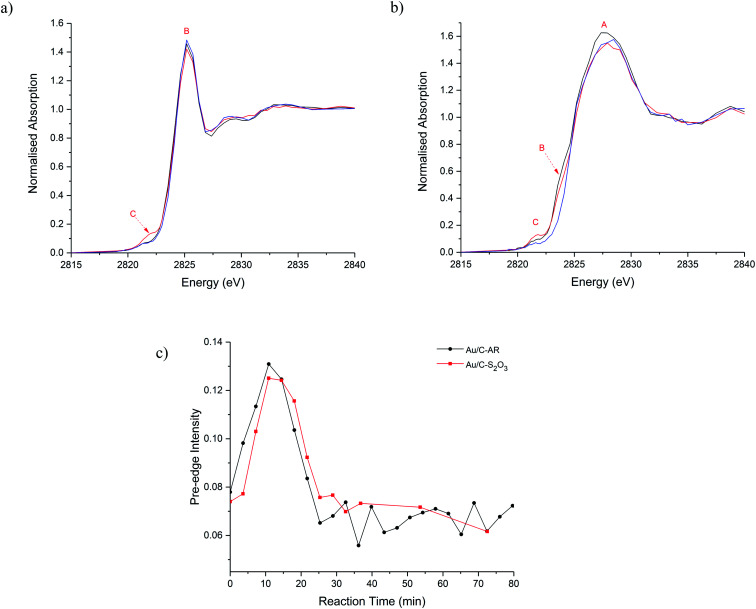
*In situ* Cl K-edge XANES spectra of (a) Au/C-AR and (b) Au/C-S_2_O_3_ catalysts during acetylene hydrochlorination at: beginning of the reaction (black line) max of pre-edge intensity (red line) and at steady state (blue line). (c) Change in pre-edge intensity with reaction time for Au/C-AR (-●-, black) and Au/C-S_2_O_3_ (-■-,red).

The catalysts were then characterised during the acetylene hydrochlorination reaction (Fig. 3a–c). Over a 100 minutes of reaction, the overall Cl concentration modestly increased for Au/C-S_2_O_3_ and was unchanged for Au/C-AR, which remained higher than the Au/C-S_2_O_3_ throughout (Fig. S8, S9 and Table S1†) which suggests that gaseous HCl cannot significantly chlorinate the carbon surface. Given the comparable VCM productivities between the catalysts (Fig. 1c),^13^ there seems to be no correlation between the total Cl concentration within the catalysts and catalytic activity. Moreover, it suggests that the Au complex and carbon surface within Au/C-S_2_O_3_ catalyst which is not highly chlorinated during the preparation is relatively resistant to possible chlorination phenomena directly from HCl and it remains significantly different to the highly chlorinated surface of the Au/C-AR. Cl(2p) XPS of the used catalysts (Fig. S10 and Table S1†) show a slight increase in Cl content in the Au/C-S_2_O_3_ catalyst after reaction, especially of the inorganic Cl species, which is attributable to a certain level of chlorination of the metal centre under reaction condition.

Comparison of the normalised Cl-edge XANES spectra (Fig. 3 and S9†), show variation in feature C (Au–Cl) during reaction in both catalysts; shown as a function of reaction time-on-line in Fig. 3c. Feature C intensifies, broadens and shifts to higher energy during the first 20 min and then decreases, suggesting changes in the Au–Cl bonding, Au oxidation state and Au–Cl_*x*_ geometry during reaction.^18^ Broadening possibly indicates multiple Au–Cl bond lengths or Au–Cl species. These observations are in line with those observed from *operando* Au L_3_-edge XAFS.^13^ The comparable changes in feature C with respect to reaction time (Fig. 3c) suggest that the Au–Cl species are similar for both catalysts. However, the significantly lower Cl concentration in Au/C-S_2_O_3_ compared to Au/C-AR throughout the reaction suggests fewer Au–Cl species in Au/C-S_2_O_3_. Given all the Au within this catalyst is cationic and atomically dispersed, this lower Cl signal shows that most of the Au is coordinated to other non-chloride ligands, most probably S ligands, throughout the reaction and that the Au in this catalyst does not become excessively chlorinated or convert to a purely AuCl_*x*_ species while operating. Under operation Au/C-S_2_O_3_ contains multiple Au speciation, a robust Au–S complex and a minority Au–Cl species analogous to Au/C-AR.

Another difference between the two catalysts Cl speciation, under reaction conditions, is the change in C–Cl species, feature B. During heating to 200 °C and during reaction this gradually reduces in intensity for Au/C-S_2_O_3_ (Fig. 3a). This behaviour can be attributed to the migration of Cl from the carbon to the Au centre, confirming the Cl(2p) XPS results on the used sample (Fig. S10†). Although the corresponding feature appears to remain stable in Au/C-AR, this is again due to the high C–Cl concentration making small differences difficult to observe. The relatively high amount and possible mobility or evolution of C–Cl species in the Au/C-AR catalyst could also explain the induction period typically observed in this type of catalyst.^13^ On heating of the catalyst, we first observe an increase in the amount of Au(iii), confirmed by both XAS of the Au L_3_-edge^13^ and Cl K-edge, before conversion to Au(i) beyond the thermal decomposition temperatures of AuCl_3_, which is approximately 130–160 °C. This observation of Au(iii) is likely the result of oxidation of Au(i) rather than the disproportionation reaction (3AuCl → 2Au + AuCl_3_) as we do not observe metallic Au in these or our previous experiments.^13^ We have previously shown that exposure to HCl alone does not result in the oxidation of Au(i) to Au(iii) species and that the induction period observed is related to the formation of Au(iii) on exposure to both reactants with activity increasing as the ratio of Au(i) to Au(iii) equilibrates. The presence of large amounts of surface chlorination could facilitate evolution of Cl_2_ from the surface at reaction temperature which is a strong enough oxidant to convert AuCl into AuCl_3_ in addition to residual NO_*x*_ species remaining from the preparation. The evolution of these species could be accelerated by the large initial exotherm passing through the bed on introduction of the reactant gases and only when the evolution of oxidant has subsided can the catalyst equilibrate to a Au(i)/Au(iii) ratio determined by catalytic turnover and steady state activity.

This behaviour is observed to a much lesser extent for Au/C-S_2_O_3_, due to the C–Cl reservoir being quickly depleted. The increased C–Cl in Au/C-AR results in a more extensive oxidation of the Au(i) chloride like species to a Au(iii) type chloride species and consequently the process of Au(i) reformation, which is correlated to activity, is considerably slower, resulting in a notable induction period as the species equilibrate.

**Fig. 4:**
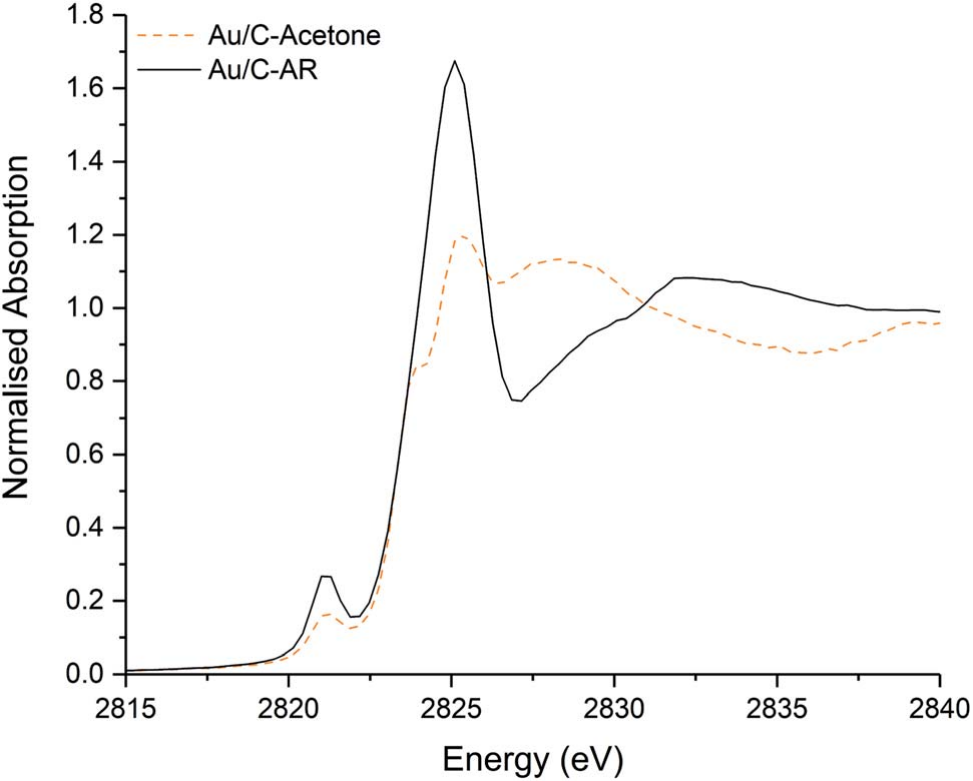
Comparison of the normalised *ex situ* Cl K-edge XANES of the freshly prepared Au/C-acetone (dashed orange line) and Au/C-AR (black solid line).

Simple impregnation of the metal precursor, as in the *aqua regia* preparation, is however preferable to the *in situ* formation of the Au–thiosulphate complex followed by its impregnation onto the carbon support required in the preparation of Au/C-S_2_O_3_. Clearly the use of an alternative non-chlorinated/organic solvent to *aqua regia* for the dissolution of the HAuCl_4_ metal precursor could be an efficient solution for the preparation of active, stable and scalable Au/C catalysts for this reaction. Recently we have shown that catalysts can be prepared using organic solvents, such as acetone.^30^ It is important to note that these catalysts which are prepared using HAuCl_4_ and without *aqua regia* (and therefore without a high Cl content) did not have an induction period and behaved analogously to Au/C-S_2_O_3_ catalysts. The Cl K-edge XANES of a catalyst prepared using HAuCl_4_ and an acetone solvent is shown in Fig. 4. Critically, the C–Cl feature is notably less intense than that seen in Au/C-AR, confirming the relationship of this species to the catalytic induction period. Although the Au/C-acetone catalyst does not have a pronounced induction period and is similar in initial activity to the Au/C-S_2_O_3_ catalyst, a proportion of the Au within the catalyst was found to reduce to form Au(0) nanoparticles. This reduction was not observed for Au/C-S_2_O_3_ catalysts, suggesting that in addition to limiting C–Cl surface species the presence of sulphur species increases the stability of the Au(i) active site.

To understand the role of sulphur-based ligands in Au/C-S_2_O_3_*in situ* S K-edge XANES experiments were also performed. The S_1s_ → S_3p_ electronic transitions are extremely sensitive to the sulphur oxidation state.^31^ Moreover, in the case of oxyanions, where the sulphur can be present in various oxidation states, the spectrum originated will show one feature (or edge) for each oxidation states of the element in the sample. Sulphur compounds have unique patterns of transitions on the absorption edge, making the sulphur K-edge XANES suitable for qualitative determination of sulphur species in samples with complex composition by using appropriate reference materials.^32,33^

**Fig. 5:**
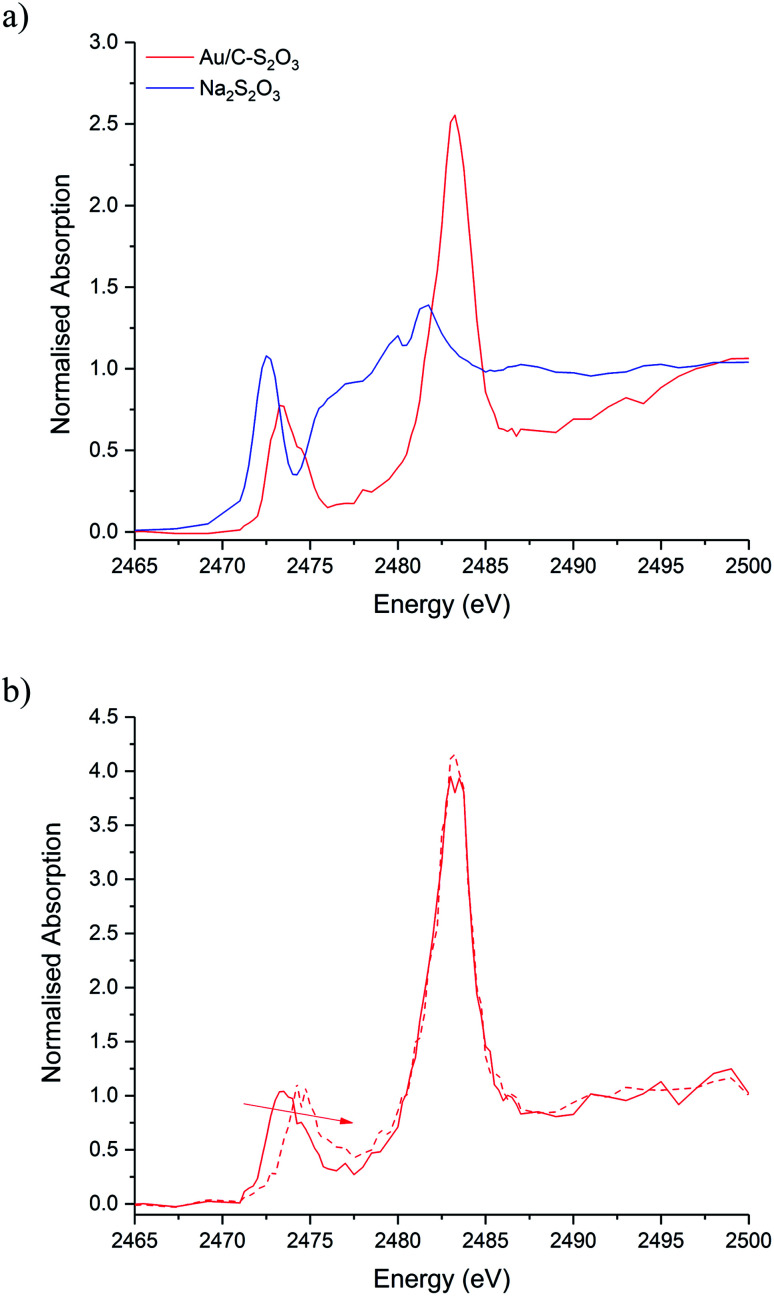
S K-edge XANES of the Au/C-S_2_O_3_. (a) *Ex situ* XANES normalised absorption spectra of Au/C-S_2_O_3_ (red solid line) with Na_2_S_2_O_3_ standard (blue solid line). (b) *In situ* XANES normalised absorption spectra during reaction time-on-line: start of reaction (red solid line) and after 20 minutes (red dashed line).

**Fig. 6:**
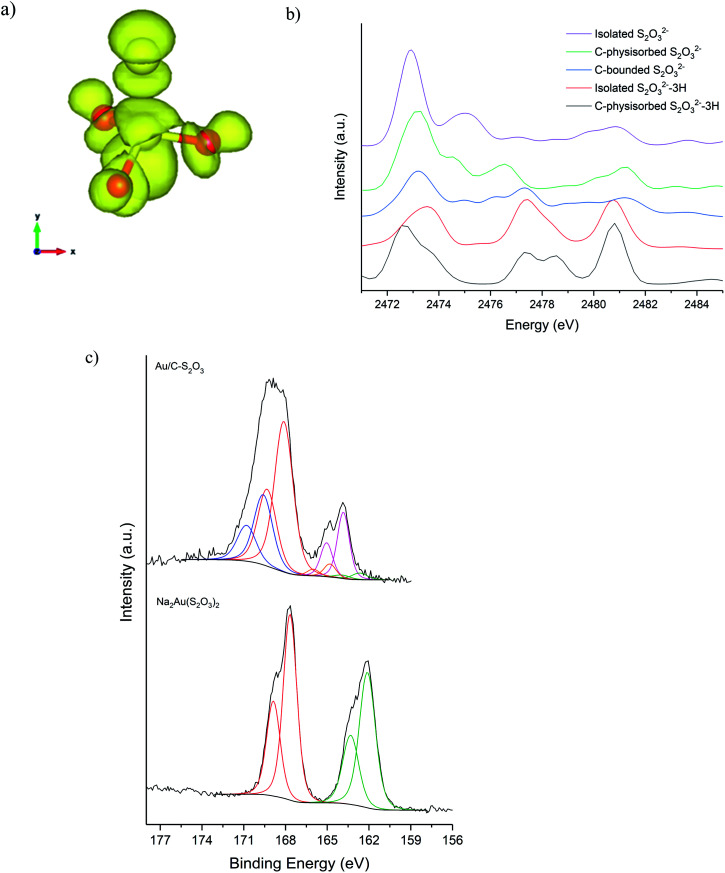
Sulphur selected orbital K-edge XANES obtained by DFT simulations. (a) Excited state responsible for transition at 2477 eV. (b) Comparison of simulated S K edge in thiosulfate S_2_O_3_^2−^ and model structures. (c) S(2p) core-level spectra for Na_2_Au(S_2_O_3_)_2_ and the Au/C-S_2_O_3_ catalyst with associated binding energy; two sulphur states in the former (S^6+^ and S^2−^ at 166.7 and 162.1 eV) and several sulphur states in the catalyst, attributed to Au–S (162.6 eV), S–S (163.8 eV), S–H (164.8 eV), SO_3_ (168.1 eV) and SO_4_ (169.6 eV), consistent with disproportionation.

Fig. 5a shows the S K-edge XANES of Au/C-S_2_O_3_ and Na_3_S_2_O_3_ (XANES spectra of other standards materials recorded are reported in Fig. S11†), where the sulphide can be differentiated from the more oxidised sulphate like species. While the formal oxidation state of sulphur in thiosulfate ions is debated,^34–36^ it is clear that species of comparable, but not identical, oxidation state are present in Na_3_S_2_O_3_ and Au/C-S_2_O_3_. Features in Na_2_S_2_O_3_ at 2472 and 2482 eV, associated with 1s → 3p transitions of the different sulphur environments, are shifted to higher energy in Au/C-S_2_O_3_ (2473 and 2483 eV) (Fig. 5a). However, features observed in Na_2_S_2_O_3_, between at 2477–2479 eV which are not satisfactorily explained in the literature, are absent in Au/C-S_2_O_3_. *Abinitio* DFT simulations of the XANES spectra of [S_2_O_3_]^2−^ show that these undefined features can be assigned to transitions to excited states where atomic orbitals of the 2 S atoms and O 2p from thiosulfate are strongly hybridized (Fig. 6a). [S_2_O_3_]^2−^ interaction with the carbon support (*via* C-π orbitals) only weakly affect these excited states (Fig. 6b). A relevant decrease of the intensity of the corresponding peaks could only be reproduced by including a strong chemical interaction in the models for the simulations (such as the addition of H covalently bonded to O of SO_3_, or the chemical binding of S_2_O_3_ to the C-based support, whose spectra are shown in Fig. 6b). The absence of these features in Au/C-S_2_O_3_ therefore suggests that the thiosulphate ligand has not remained intact on deposition onto the carbon and has potentially undergone a disproportionation reaction to S^2−^ and SO_3_^2−^. Furthermore, the XANES simulations of the model structures where a strong chemical interaction is consistently included, also successfully predict an increase in the energy of the first electronic transition to 2473 eV consistent with the shift of the first peak observed in experimental spectra shown in Fig. 5 between Na_2_S_2_O_3_ and Au/C-S_2_O_3_.

XPS analysis of the Au/C-S_2_O_3_ catalyst supports this supposition. Fig. 6c shows the S(2p) region for Na_3_Au(S_2_O_3_)_2_ and Au/C-S_2_O_3_; with two sulphur states in the former (S^6+^ and S^2−^ at 166.7 and 162.1 eV) and several additional sulphur states in the catalyst, attributed to Au–S (162.6 eV), S–S (163.8 eV), S–H (164.8 eV), SO_3_ (168.1 eV) and SO_4_ (169.6 eV), consistent with disproportionation. The presence of Au–S bonds in Au/C-S_2_O_3_ proposed based on XPS binding energy and splitting of the XANES S^2−^ feature, as seen in Na_3_Au(S_2_O_3_)_2_ (Fig. S11†), confirms that the Au is predominantly bound to the sulphide species. To understand the dynamic nature of S within Au/C-S_2_O_3_, *in situ* S K-edge XANES studies were performed. During heating to reaction temperature under an inert atmosphere, the two main absorption edges did not change in intensity or position (Fig. S12†), in contrast with Cl speciation. Demonstrating that S speciation in Au/C-S_2_O_3_ was more thermally stable than the Cl species.

Introduction of reaction gases resulted in a shift in the absorption-edge of the S^2−^ bound to Au, during the first 20 min of reaction, while the SO_3_^2−^ group remains stable (Fig. 5b and S13†). The shift to higher absorption-edge for the sulphide species shows a decrease in electron density and oxidation of this S species bound to Au. We suggest that Au(i) oxidation, caused by C–Cl surface species on the carbon is suppressed by the polarisable Au–S bond. Therefore, in addition to the significantly lower concentration of C–Cl species, the active Au(i) species in Au/C-S_2_O_3_ do not over oxidise and the catalyst is immediately active. This supports the proposed detrimental role of the Cl excess on the carbon surface. The S 2p XPS of the Au/C-S_2_O_3_ catalysts before and after reaction (Fig. S14 and Table S4†) show that multiple sulphur states can be observed as in Fig. 6c with identical speciation assignments. It should be noted however, binding energies for the species differ by *ca.* 0.2–0.6 eV, the smaller difference attributable to experimental errors and uncertainty in peak fits whilst the larger difference may again be attributed to agglomeration of sulphur species and/or higher uncertainty in the peak fits due to the signal to noise in the data.

**Fig. 7:**
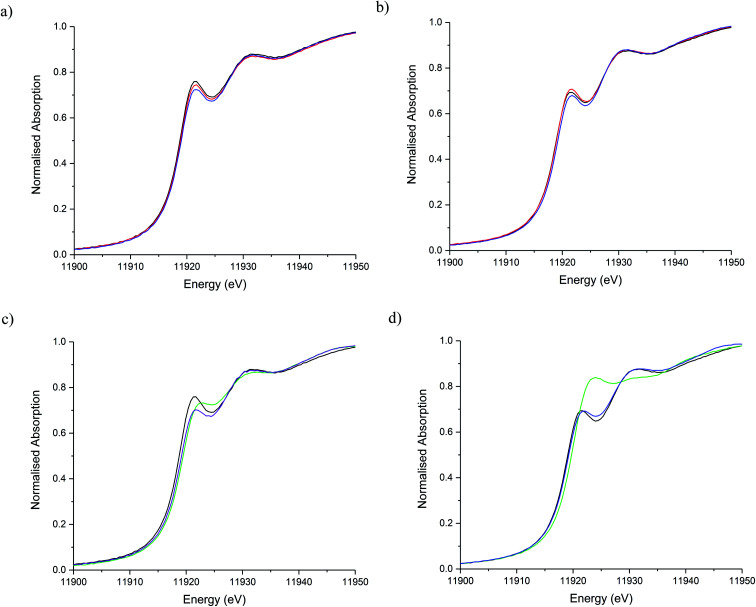
Au L_3_-edge XANES of Au/C-S_2_O_3_ and Au/C-AR during the sequential gas exposure experiments. (a) Au/C-S_2_O_3_ and (b) Au/C-AR at steady state for steps i (red line), steps ii (blue line) and steps iii (black line). (c) Au/C-S_2_O_3_ and (d) Au/C-AR at steady state for steps iii (black line), steps iv (green line) and steps v (purple line).

**Fig. 8:**
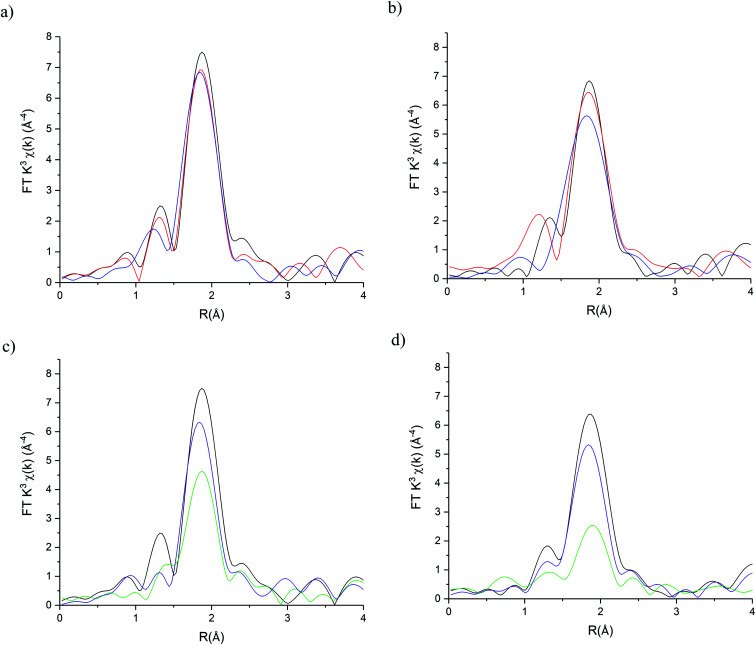
Au L_3_ edge EXAFS Fourier Transform magnitudes of Au/C-S_2_O_3_ and Au/C-AR during the sequential gas exposure experiments. (a) Au/C-S_2_O_3_ and (b) Au/C-AR at steady state for steps i (red line), steps ii (blue line) and steps iii (black line). (c) Au/C-S_2_O_3_ and (d) Au/C-AR at steady state for steps iii (black line), steps iv (green line) and steps v (purple line).

The stability of the S containing Au/C-S_2_O_3_ catalyst was investigated at the L_3_-edge using an “accelerated deactivation test”, in which the catalyst is sequentially exposed to; (i) HCl + C_2_H_2_, (ii) HCl only, (iii) HCl + C_2_H_2_ (iv) C_2_H_2_ only and finally (v) HCl + C_2_H_2_ during one experiment. We have already reported that exposure to HCl only (*i.e.* in the absence of C_2_H_2_) resulted in Au/C-AR catalytic performance being perturbed on the re-introduction of C_2_H_2_, resulting in a second induction period to regain steady state activity.^1,37^ Also, Au/C-AR rapidly deactivates after treatment with only C_2_H_2_.^37^ This deactivation was attributed to an interaction with AuCl_*x*_ and C_2_H_2_ that on the re-introduction of HCl causes the formation of Au(0) nanoparticles. To clarify the influence of the individual reactants on the Au/C-S_2_O_3_, the VCM productivity (Fig. S15†) and the Au L_3_-edge XANES (Fig. 7) and EXAFS (Fig. 8 and Table 1) were correlated during an *operando* experiment in a fixed bed reactor. XANES and EXAFS spectra of the Au/C-AR catalyst, reported previously,^37^ are also shown for comparison. These previous studies show that under steady state reaction conditions a combination of Au(i) and Au(iii) chloride/sulphur compounds are present, with EXAFS determined coordination numbers matching those determined from XANES analysis, by white line height or linear combination fitting.^13,37^

**Table 1:** EXAFS Fitting for Au L_3_-edge data of Au/C-S_2_O_3_ and Au/C-AR catalysts during sequential gas experiments

Reaction step^a^	Scattering path^b^	Coordination number		2*σ*^2^ (Å^2^)^e^		*R* (Å)		*E*_f_ (eV)		*R* factor	
		Au/C-S_2_O_3_	Au/C-AR^c^	Au/C-S_2_O_3_	Au/C-AR	Au/C-S_2_O_3_	Au/C-AR^c^	Au/C-S_2_O_3_	Au/C-AR^c^	Au/C-S_2_O_3_	Au/C-AR^c^
i	Au–Cl	2.5(1)	2.5(1)	0.0037		2.27(1)	2.27(1)	2(1)	1(1)	0.011	0.012
ii	Au–Cl	2.5(1)	2.4(1)			2.27(1)	2.27(1)	1(1)	3(1)	0.012	0.07
iii	Au–Cl	2.6(2)	2.5(1)			2.28(1)	2.27(1)	2(1)	3(1)	0.022	0.04
iv (model 1)^f^	Au–Cl	2.2(3)	2.0(4)	0.006(2)	0.011(2)	2.28(1)	2.29(2)	1(2)	1(2)		0.031
iv (model 2)	Au–Cl (1)	1.6(1)	0.9(2)	0.0037		2.28(2)	2.30(3)	1(1)	1(4)	0.020	0.037
	Au–C^d^	1	1	0.0090		2.28(1)	2.04(7)				
v	Au–Cl	2.2(1)	1.7(3)	0.0037		2.27(1)	2.26(1)	1(1)	2(2)	0.019	0.017
	Au–Au^d^	1.0(4)	0.6(4)	0.007		2.88(2)	2.84(5)				
	Fixed parameters: *S*_o_^2^ = 0.75, 2*σ*^2^ (Å^2^) = 0.0037 (Au–Cl) and 0.007 (Au–Au)										

a EXAFS data taken under steady state conditions.

b Fitting of multiple Au–Cl paths was attempted for all spectra. Only is step iv did this provide realistic fits.

c Au/C-AR data published in ref. 37.

d Au–C CN fixed at 1.

e Debye–Waller and amplitude reduction factors were determined from fitting of KAuCl_4_ with a fixed CN of 4 or Au(0) with fixed CN of 12.

f Debye–Waller fitted for model 1 (step iv).

After a small drop in productivity over the first 20 min time-on-line Au/C-S_2_O_3_ remained stable for the complete test duration with minimal deactivation despite periods of being exposed to only HCl or C_2_H_2_. The XANES spectra of Au/C-S_2_O_3_ showed a slight decrease in white-line height, as seen for Au/C-AR, under the HCl environment. It is clear that for both catalysts the Au(iii/i) centres are not strongly influenced by the dilute HCl stream and that neither catalyst is oxidised to a higher degree of chlorination under these conditions. This is corroborated by the EXAFS which shows no change in the Au–Cl/S (indistinguishable by EXAFS) coordination number of 2.5 or bond lengths between reaction (i) and under HCl (ii).

The difference in catalyst performance on re-introduction of both reactants following HCl treatment (step iii) between Au/C-S_2_O_3_ and Au/C-AR is therefore due to the C–Cl content on the two respective support structures. Namely, the absence of significant C–Cl content in Au/C-S_2_O_3_ resulting in an immediate return to reactivity, without an induction period. Under a C_2_H_2_ flow, the energy of the absorption edge increased for Au/C-S_2_O_3_, with the observed white-line shifting from 11 921.6 eV to 11 922.7 eV. Comparison with the change in XANES seen for Au/C-AR under identical C_2_H_2_ conditions showed similar trends to that observed for Au/C-S_2_O_3_. The change in white-line for both catalysts is attributed to an uncharacterised Au–acetylene interaction.^37^ However, the extent of the edge shift was significantly less for Au/C-S_2_O_3_ than that seen for Au/C-AR. The Fourier transformed Au L_3_-edge EXAFS (Fig. 8) showed a decrease in the magnitude of the signal at 1.8 Å for both catalysts. Again, the dampening of the signal was notably less for Au/C-S_2_O_3_ under C_2_H_2_ relative to Au/C-AR.

The damping of oscillations seen for Au/C-AR was previously attributed to out of phase multiple Au–Cl path lengths.^37^ The catalyst was considered to be Au(i) based on the edge position but with a significant change in geometry. The rational for this interpretation being that *χ* space data showed a damping of oscillations across the *k* range under C_2_H_2_, with no change in the phasing between a catalyst under C_2_H_2_ (step iv) or under steady state reaction conditions (step i–iii) (Fig. S16†), suggestive of no new scatter being present and an increase in disorder. However, an unresolved question of the cause of this affect, in addition to the significant change in the XANES of both the Au/C-AR and Au/C-S_2_O_3_ catalysts under C_2_H_2_, prompts consideration of an alternative interpretation of the data. A compelling hypothesis being that in addition to Au–Cl paths there is a Au–C path attributable to Au–C_2_H_2_ bonding. Therefore, the fitting of the EXAFS data of Au/C-AR and Au/C-S_2_O_3_ catalysts under C_2_H_2_ was performed using two models; (1) a single Au–Cl path with a floated 2*σ*^2^ parameter to account for an increased structural disorder due to multiple Au–Cl paths or (2) fitting Au–Cl and Au–C paths.

Both models provided reasonable fits of the data. Model 1 showed a significant increase in 2*σ*^2^ from 0.0037 Å^2^ under steady state (step i) to values under C_2_H_2_ of 0.006(2) Å^2^ and 0.011(2) for Au/C-AR and Au/C-S_2_O_3_ respectively. Model 2 provides a viable Au–C path length of 2.04(7) Å which, despite the significant error, fits with distances seen for Au(i) σ-bonded alkyne complexes at 1.98 Å.^38^ Freeing of the Au–Cl/S coordination number showed that this path was more significant in Au/C-S_2_O_3_ and suggest that more free Au(i) is present in this catalyst than Au/C-AR. Comparison of the fits shows, however, no statistical improvement on the addition of a Au–C path, further this model significantly misrepresented the first oscillation of the *χ* space data where a soft scatterer would be best represented (low *k* distances, Fig. S17†). It is concluded, that whist compelling to evoke Au–alkyne complex formation it could not be definitively proven with the current data. Regardless of the fitting model used it is clear that C_2_H_2_ has a similar influence on Au/C-S_2_O_3_, although it is far less dramatic than that seen for Au/C-AR.

The effect of reintroduction of both reactant gases in step v on Au speciation was notably different for the two catalysts. While Au/C-AR was found to deactivate after the re-introduction of HCl, Au/C-S_2_O_3_ remained relatively stable with only minimal deactivation. As stated previously, the growth of Au(0) responsible for deactivation in Au/C-AR, occurred only after re-introduction of HCl (step iv) and not under C_2_H_2_.^37^ Au–Au paths could be fitted for both catalysts at the end of reaction step iv, at all other points during the reaction no acceptable fit of this path was found. The proportion of Au(0) could not accurately be determined by EXAFS due to the large error in Au–Au coordination numbers.

## Conclusion

S and Cl K-edge XANES have been used to gain further insight into the structure–activity relationship of cationic Au catalysts in acetylene hydrochlorination. Three Cl species; unreactive inorganic Cl, covalently bound C–Cl and Au–Cl species are found on Au/C-AR and Au/C-S_2_O_3_. The concentration of these species was significantly higher in Au/C-AR. Au–Cl species are dynamic during heating and under reaction conditions, with changes in Au–Cl bond hybridisation and bond length. In addition, surface bound C–Cl is found to be titrated from the carbon surface during reaction suggesting the potential importance of these surface groups in influencing Au–Cl speciation. High concentrations of C–Cl, as seen in Au/C-AR, therefore result in a catalytic induction period. The presence of Au–Cl in Au/C-S_2_O_3_ was concluded to be from a minority phase that coexists with Au–S species. The presence of intact [S_2_O_3_]^2−^ ligands could not be confirmed on deposition of the Au complex onto the carbon support. However, the S species present were found to be stable under reaction conditions and stabilises Au(i) to produce a robust and commercially viable acetylene hydrochlorination catalyst.

## Experimental

### Catalysts preparation

1 wt% gold supported on activated carbon catalysts were prepared by wet impregnation of the HAuCl_4_ precursor dissolved in *aqua regia* or water (denoted as Au/C-AR and Au/C-H_2_O respectively). Activated carbon was initially ground to obtain a 100–140 mesh powder. The gold precursor, HAuCl_4_·*x*H_2_O (Alfa Aesar, 99.9% (metals basis), Au 49%) was dissolved in *aqua regia* (3 parts by volume HCl [(Fisher, 32 wt%)]: 1 part by volume HNO_3_ [(Fisher, 70 wt%)]) or water. The gold precursor solution was then added drop-wise with stirring to the acid washed, steam activated wood carbon. Stirring was continued at ambient temperature for 1 hour or until NO_*x*_ production subsided. The product was then dried for 16 h at 140 °C under an inert flow of nitrogen. A different procedure was employed for the preparation of the 1 wt% Au/C-S_2_O_3_ catalyst: an aqueous HAuCl_4_·3H_2_O solution was mixed with an aqueous solution of sodium thiosulfate, Na_2_S_2_O_3_, in order to obtain a NaAuS_2_O_3_ complex. The mixture obtained was added slowly in aliquots to the support while stirring. The product was then dried at 110 °C overnight under a nitrogen flow.

### Catalysts testing

#### *In situ* K-edge XANES experiment

A microreactor was located inside an environmental chamber or vacuum vessel that allow a vacuum or a helium atmosphere. Thus, specifics microreactor (flow cell) and reaction conditions have been used. The set-up (vacuum vessel and cell) used have been developed by Thompson and Newton and it is widely described elsewhere in the literature.^39^ The flow cell is placed in the core of the environmental chamber.

Within this setup the dedicated space for the catalyst bed is very limited and the amount of catalyst used was *ca.* 10 mg. For this reason, the total flow has been reduced to 5 mL min^−1^: C_2_H_2_/He (2.5 mL min^−1^) HCl/He (2.5 mL min^−1^). Dilute gas mixtures were used C_2_H_2_/He (4.97% balanced in He, Air Liquide) and HCl/He (5.00% balanced in He, Air Liquide). The gases were dried, using moisture traps, prior to introduction to the reactor setup. In all cases, the reactor was purged with He (99.99%, Air Liquide), heated to 200 °C at a ramp rate of 2.5 °C min^−1^ and held at temperature for 30 min, all under a flow of He (5 mL min^−1^), prior to admitting the hydrochlorination reaction mixture. The outlet gas line was connected to a mass spectrometer (Hiden QGA), to detect VCM during reactions and showed that the catalysts were functioning.

#### *Operando* Au L_3_-edge XAS and laboratory activity test

The catalysts were tested using a completely automated reactor system with the same setup as previously described.^13^ All of the predilute gases 5% C_2_H_2_/Ar (BOC), 5% HCl/Ar (BOC), and Ar (99.99% BIP, Air Products) were dried using moisture traps before being introduced into the reactor. In all cases the reactor was heated to 200 °C at a ramp rate of 5 °C min^−1^ and held at this temperature for 30 min under a flow of argon prior to admitting the hydrochlorination reaction mixture. The tests were performed using a fixed-bed polyimide (Kapton) microreactor containing the catalysts, keeping the total flow of 50 mL min^−1^ and a total gas hourly space velocity (GHSV) of ∼14 000 h^−1^. When both reactants were present, the C_2_H_2_ : HCl ratio was kept at a constant value of 1 : 1.02.

The reaction mixture was analysed on-line by mass spectrometry (Hiden QGA), and Professional Edition software was used for both qualitative and quantitative analyses. The catalyst activity presented is shown in terms of productivity toward vinyl chloride monomer (VCM). The response factor of the mass spectrometer toward VCM was correlated with the productivity (mol kg_cat_^−1^ h^−1^) obtained by using a Varian 450 gas chromatograph equipped with a flame ionization detector (FID). Chromatographic separation and identification of the products was carried out using a Porapak N packed column (6 ft × 1/8′′ stainless steel).

The sequential flow experiment was performed simultaneously monitoring the Au L_3_-edge XAS and catalytic activity. Reaction sequence employed the following gas compositions: step i = HCl/C_2_H_2_/Ar, step ii = HCl/Ar, step iii = HCl/C_2_H_2_/Ar, step iv = C_2_H_2_/Ar, and step v = HCl/C_2_H_2_/Ar. The duration of each step in the sequence was not the same. The gas composition during the experiment was altered only when no further change in the XAS spectra was observed.

### Catalysts characterisation

#### X-ray absorption spectroscopy (XAS)

*In situ* and *ex situ* XANES spectra have been acquired at the BM28 (XMaS) beamline at the European Synchrotron Radiation Facility (ESRF), situated on the soft end of an ESRF dipole magnet, which provides a wide range of X-ray techniques making use of white beam and monochromatic energies in the range of 2.4 to 15 keV. At the BM28 it is possible to run a so-called XESCAN.MAC – Extended Escan: Variable point density. In particular, it is possible to perform energy scan for multiple consecutive energy regions, with equal or different step (variable point density). The sample chamber was purged with helium for at least an hour to remove air introduced during sample loading. The beam upstream of the sample chamber is contained within a helium-filled tube to minimise X-ray absorption by the air. The fluorescent signal was detected using a silicon drift diodes detector. All spectra have been acquired in fluorescence mode.

*In situ* and *ex situ* XAFS at the Au L_3_-edge and Cl K-edge have been also acquired at the B18. The Cl K-edge X-ray absorption spectra (XAS) have been measured to probe chloride-gold bonding. Spectra for the Au/C-AR samples at different time-on-line were recorded *ex situ* at the Cl K absorption edge in fluorescence mode, using beamline B18 of the Diamond Light Source, Harwell, UK. The measurements were performed using a QEXAFS setup with a fast-scanning Si (111) double crystal monochromator and a 36 element Ge fluorescence detector. The K-edge absorption spectrum of Cl when bound to a transition metal shows a pre-edge feature due to the forbidden 1s → 3d transition. This transition becomes partially allowed and therefore observed when the Cl p-orbitals mix with the metal d-orbitals. The position of the pre-edge feature is dependent on several factors, namely (i) the Cl 1s energy, related to the charge on the chloride, and (ii) the metal d-orbital energy which is itself determined by both the oxidation state of the metal and the coordination number. The intensity of the pre-edge feature is dependent on the mixing of the Cl orbitals and metal d-orbitals and so the bonding characteristic of the Cl to the metal.

#### X-ray photoelectron spectroscopy (XPS)

XPS was carried out using a Thermo Scientific K-alpha photoelectron spectrometer with microfocused monochromatic Al K_α_ radiation operating at 72 W (6 mA × 12 kV), the value of the C(1s) peak under the operating conditions was found to be 284.5 eV, typical of activated carbons with graphic character as per carbon supports used here. The resulting spectra were processed in CasaXPS (v2.3.21) using a Shirley type background removal, Scofield cross-sections and an electron energy dependence of −0.6.

#### DFT simulations

Within the framework of DFT simulations, we used the software package ORCA v4.1 (ref. 40) and compared two levels of DFT theory, including generalised-gradient functionals (BP86 and PBE)^41^ and hybrid functionals (B3LYP),^42^ obtaining comparable spectral simulations for the S K edge.

We tested the consistency of the results obtained with 6-311G*, triple-ζ split-valence plus polarization and double-ζ split-valence basis set for both S and O atoms. Here we show the results obtained with def2-TZVP basis set. Geometries of the molecules were relaxed within an energy tolerance of 5.0 × 10^−6^ a.u. and a maximum displacement of 4.0 × 10^−3^ a.u. The electronic properties of the ground state were converged to an energy threshold of 1.0 × 10^−7^ a.u. Sulphur K edge spectra were then calculated within the TD-DFT formalism for the excited states.^43^ A rigid shift has been applied consistently among all the spectra shown in Fig. SIM1† to align them at the energy of the experimental S K absorption spectra.

## Conflicts of interest

There are no conflicts of interest to declare.

## Supplementary Material

SC-011-D0SC02152K-s001
