# Supplementary material for: In situ K-edge X-ray absorption spectroscopy of the ligand environment of single-site Au/C catalysts during acetylene hydrochlorination
Source: Chem Sci. 2020 Jun 24;11(27):7040–52. doi: 10.1039/d0sc02152k (PMC8159275; doi:10.1039/d0sc02152k)
Supplement: SC-011-D0SC02152K-s001 [file SC-011-D0SC02152K-s001.pdf]

Electronic Supplementary Material (ESI) for Chemical Science.
This journal is © The Royal Society of Chemistry 2020

Supplementary Information

In situ K-edge X-ray absorption spectroscopy of the ligand environment of single-site Au/C catalysts during acetylene hydrochlorination.

Grazia Malta[1], Simon A. Kondrat,[2] Simon J. Freakley,[3] David J. Morgan,[1] Emma K. Gibson[4,5], Peter P. Wells,[6,7] Matteo Aramini[7], Diego Gianolio[7], Paul B. J. Thompson[8,9], Peter Johnston[10] and Graham J. Hutchings[1]*

1) Cardiff Catalysis Institute, School of Chemistry, Cardiff University, Main Building, Park Place, Cardiff, CF10 3AT, UK.

2) Department of Chemistry, Loughborough University, Loughborough, Leicestershire, LE113TU, U.K

3) Department of Chemistry, University of Bath, Bath, BA2 7AY, U.K.

4) School of Chemistry, Joseph Black Building, University of Glasgow, Glasgow G12 8QQ, U.K.

5) UK Catalysis Hub, Research Complex at Harwell, RAL, Oxford, OX11 0FA, UK.

6) School of Chemistry, University of Southampton, Southampton, SO17 1BJ, U.K.

7) Diamond Light Source, Harwell Science and Innovation Campus, Chilton, Didcot OX11 0DE, UK.

8) XMaS, UK CRG, ESRF, 71 Avenue des Martyrs, 38043 Grenoble, France

9) Department of Physics, University of Liverpool, Oliver Lodge Laboratory, Liverpool L69 7ZE, UK

10) Process Technologies, Johnson Matthey PLC, Billingham, TS23 1LB, UK.

*Hutch@Cardiff.ac.uk

Table S1. Chlorine content in the catalysts, fresh and used, obtained from the raw Cl K-edge XANES and Cl(2p) core-level XPS and spectra.

Catalyst	Cl K-edge (XANES) (a.u.)	Cl 2p (XPS) [at%]
Au/C-AR Fresh	0.29	1.98
Au/C-AR Used	0.21	1.75
Au/C-S_2O_3 Fresh	0.08	0.25
Au/C-S_2O_3 Used	0.09	0.42
Au/C-H_2O Fresh	0.03	0.28

Table S2. Chlorine content in the carbon support, bare and after treatment with H_2O and *aqua regia*, obtained from the Cl(2p) core-level XPS and raw Cl K-edge XANES spectra. (Relative spectra are reported in figure S5 and S6).

Catalyst	Cl K-edge (XANES) (a.u.)	Cl 2p (XPS) [at%]
C	0.01	0.08
C-H_2O	0.007	-
C-AR	0.22	1.90

Table S3. Oxygen content obtained from the O(1s) core-level XPS spectra.

Catalyst	O 1s (XPS) [at%]
Au/C-AR Fresh	6.27
Au/C-AR Used	6.47
Au/C-S_2O_3 Fresh	4.85
Au/C-S_2O_3 Used	4.13
C	3.75
C-H_2O	3.49
C-AR	6.68

Table S4. Sulphur content obtained from the *ex situ* recorded raw S K-edge XANES spectra and S(2p) core-level XPS spectra.

Catalyst	S K-edge (XANES) (a.u.)	S 2p (XPS) [at%]
Au/C-S_2O_3 Fresh	$1.7 \cdot 10^{-3}$	0.64
Au/C-S_2O_3 Used	$0.9 \cdot 10^{-3}$	0.43

Figure S1. *Ex situ* Cl K edge XANES of Au/C-AR (-■-), Au/C-S_2O_3 (-●-) and Au/C-H_2O (-▲-).

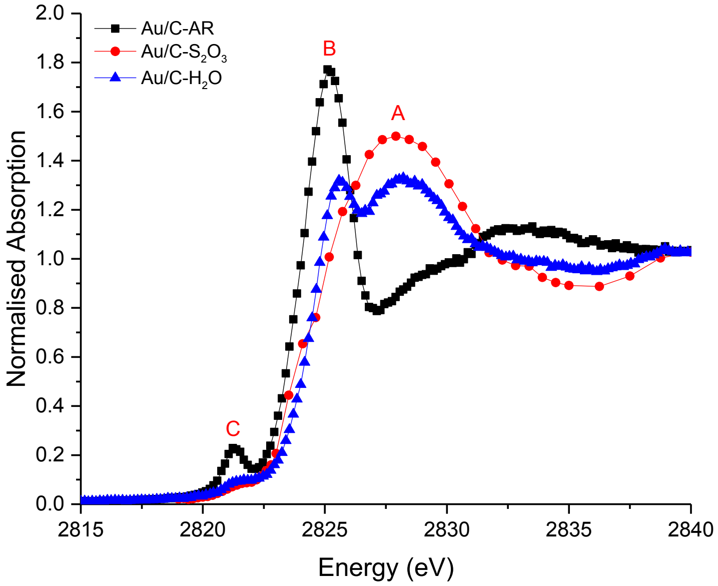

Figure S2. Comparison between raw (-■-) and normalised (-▲-) XANES spectra for the freshly prepared a) Au/C-AR and b) Au/C-S_2O_3.

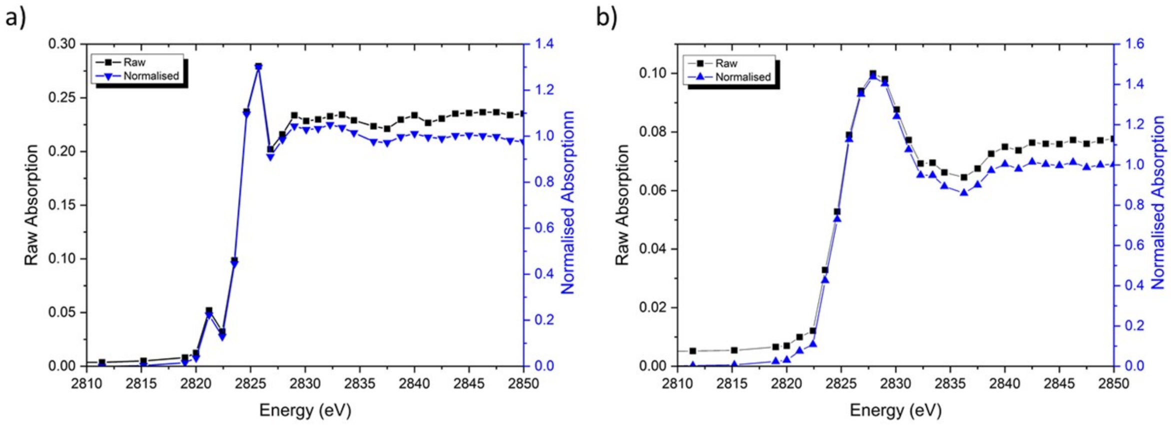

All the spectra acquired (*ex situ* and *in situ*) have been initially processed using “PyMca”, which has been developed by the Software Group of the European Synchrotron Radiation Facility (ESRF). [1] In particular, all the raw fluorescence spectra which have been initially standardised by the incident beam, and then converted from ‘.mca’ to ‘.dat’ called “Raw fluorescence absorption”. Comparisons have been made between raw fluorescence spectra recorded at the same detector position, i.e. *detin* = 99.0. Moreover, when comparing the Cl absorption of the raw fluorescence spectra, the absorption values have been taken at a fixed energy of 2.85 keV.

The pre-edge energy position, intensity and shape have been used to provide information on the character of the bond and associated coordination geometry of the Au-Cl complex.[2] Conventional data reduction [3] of the XANES spectra have been performed using Demeter Software package [4] to obtain the so-called “normalised” spectra.

Figure S3. *Ex situ* Cl 2p XPS of Au/C-H_2O catalyst. The first two peaks are associated with "inorganic" Cl (~197 eV), while the other two peaks are associated with "organic" Cl (~200 eV).

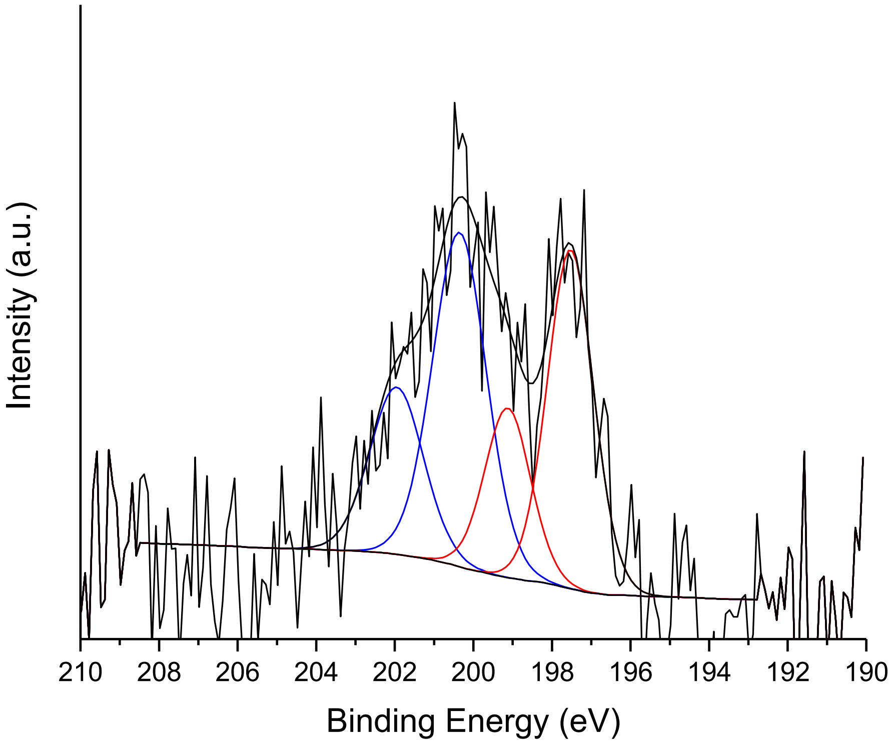

Figure S4. Cl K edge XANES of Au/C-AR (-■-), Au/C-S_2O_3 (-●-), Au/C-H_2O (-▲-) and the reference material, NaCl (-◆-).

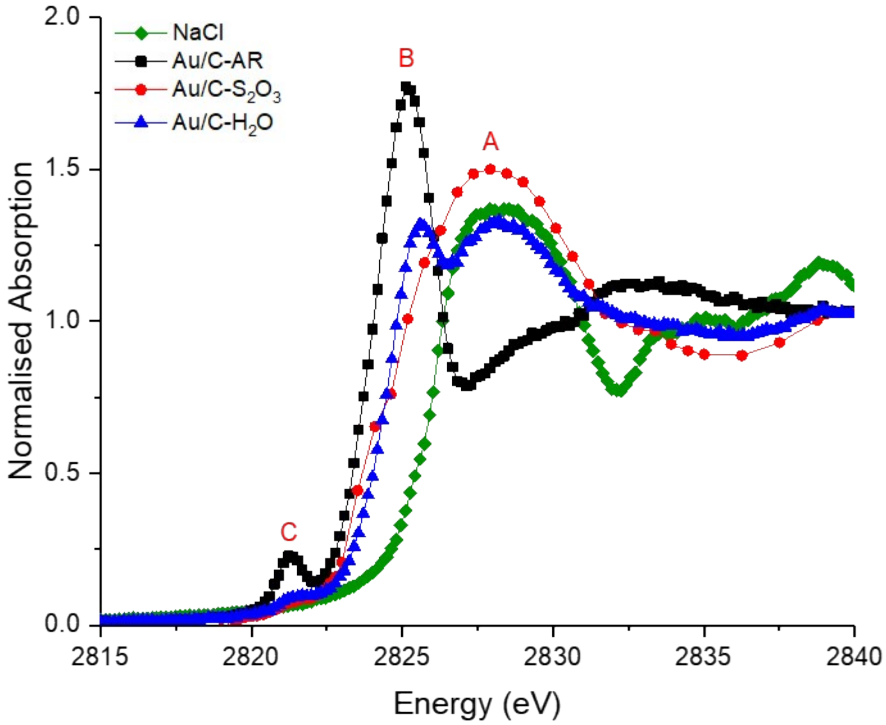

Figure S5. Cl K edge XANES of bare carbon, C (-▲-) carbon washed with aqua regia, C-AR (-●-) and carbon washed with water (-■-). See Table 2 for quantification of the Cl content.

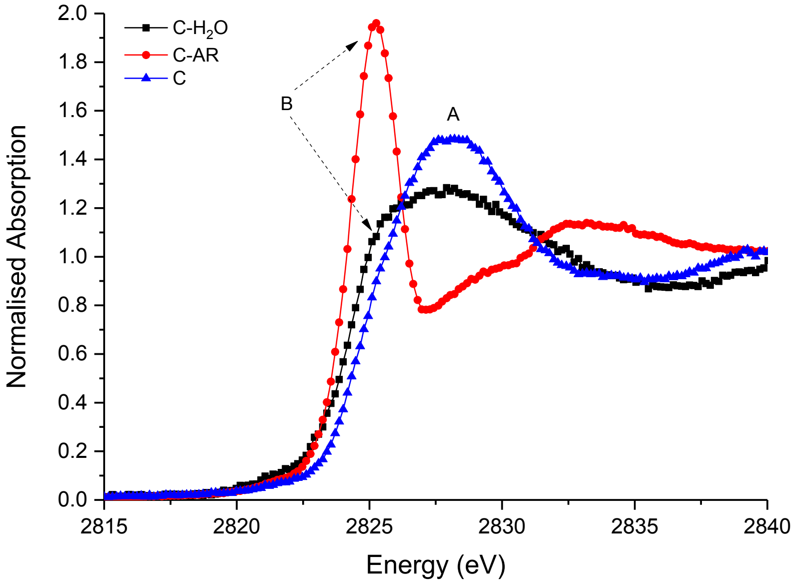

Figure S6. *Ex situ* Cl 2p XPS of a) the bare carbon and treated with b) water, C-H_2O and c) *aqua regia*, C-AR. See table 2 for quantification of the Cl content.

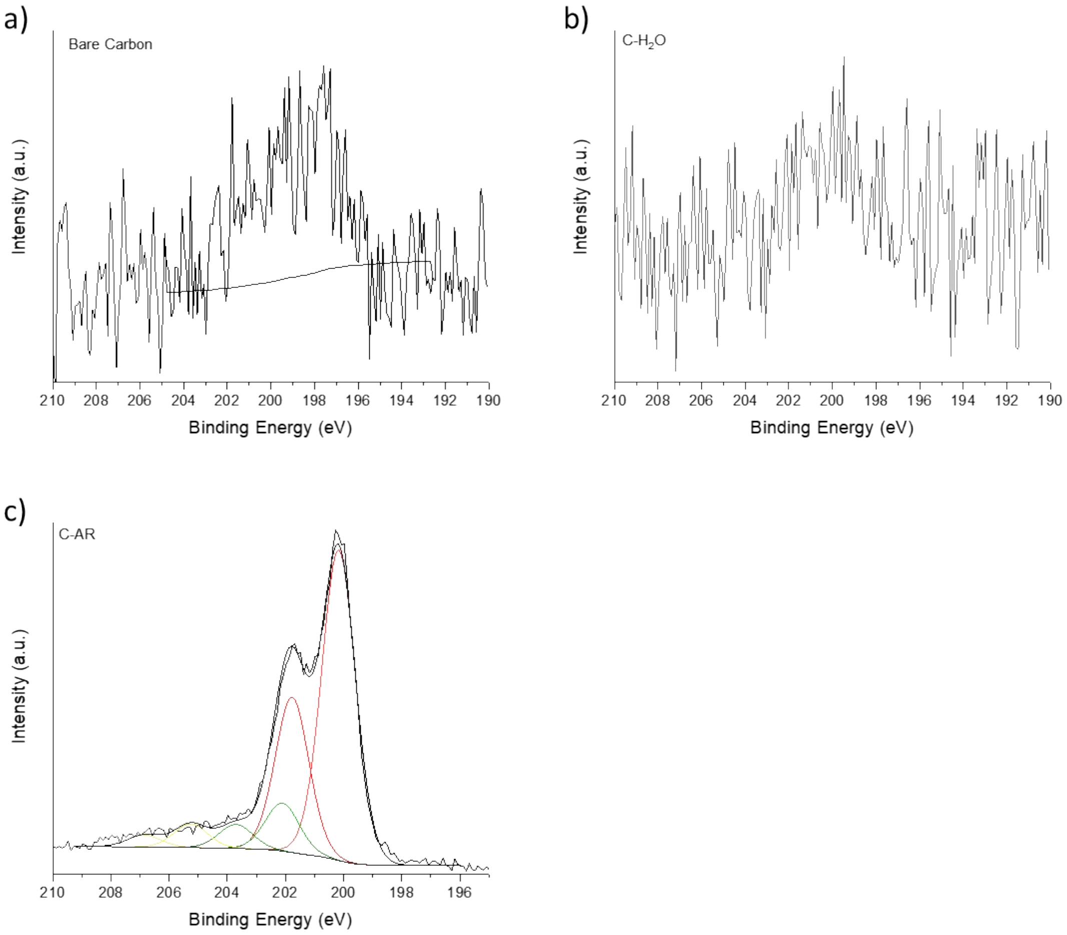

Figure S7. All Cl K edge XANES during heating to 200 °C under helium of a) Au/C-AR and b) Au/C-S_2O_3 catalysts.

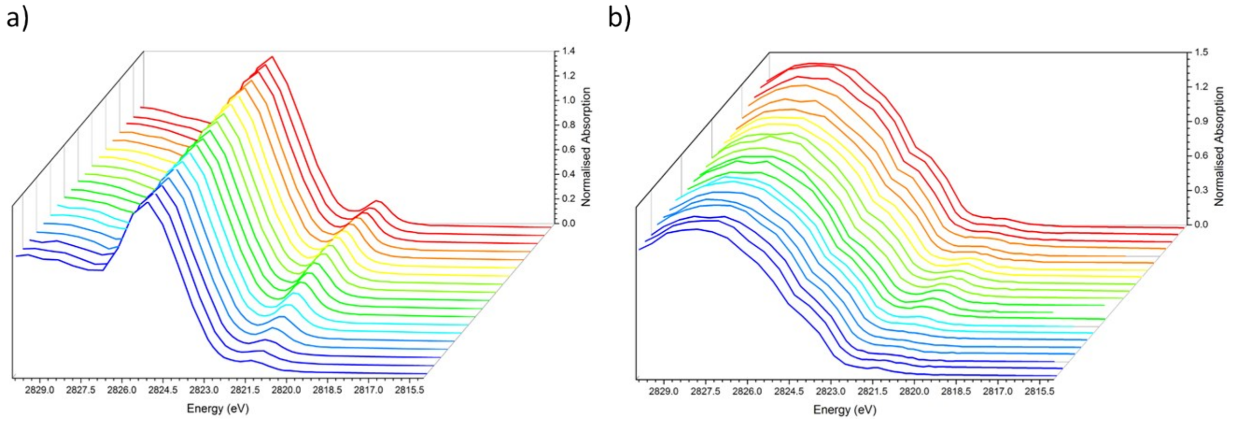

Figure S8. All Cl K edge normalised XANES during the time-on-line of the a) Au/C-AR and b) Au/C-S_2O_3 catalysts.

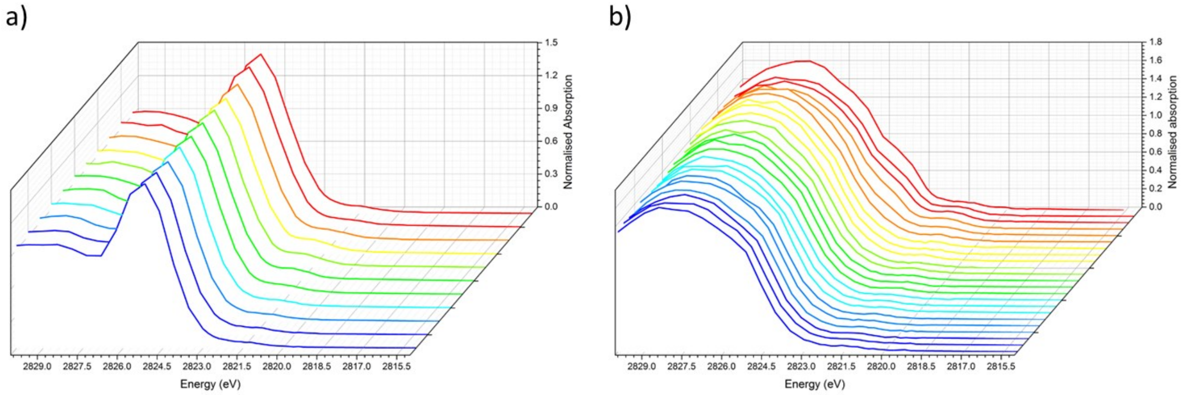

Figure S9. Comparison between raw XANES spectra at the beginning (-■-) and at the end of the reaction time (-●-) of a) Au/C-AR and b) Au/C-S_2O_3 catalysts.

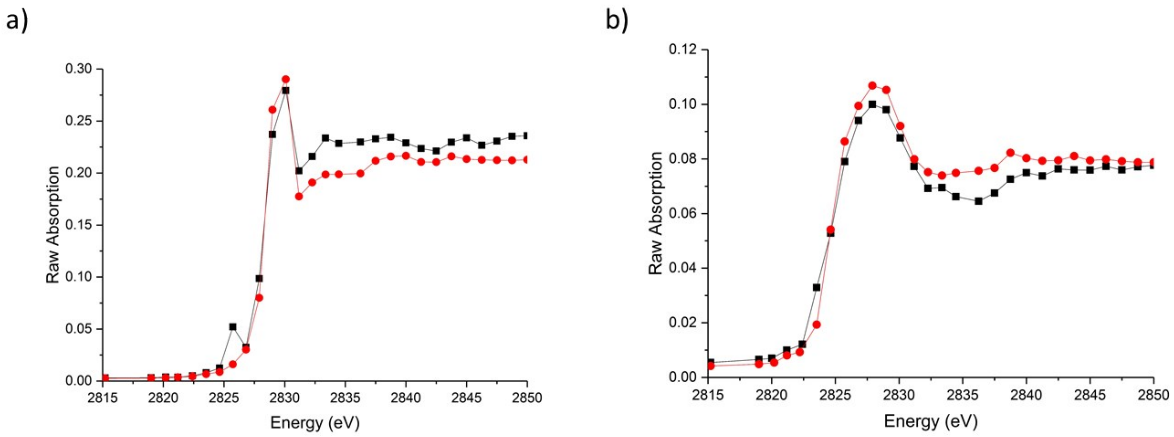

Figure S10. *Ex situ* Cl 2p XPS of the used a) Au/C-AR and b) Au/C-S_2O_3 catalysts.

See table S1 for quantification of the Cl content.

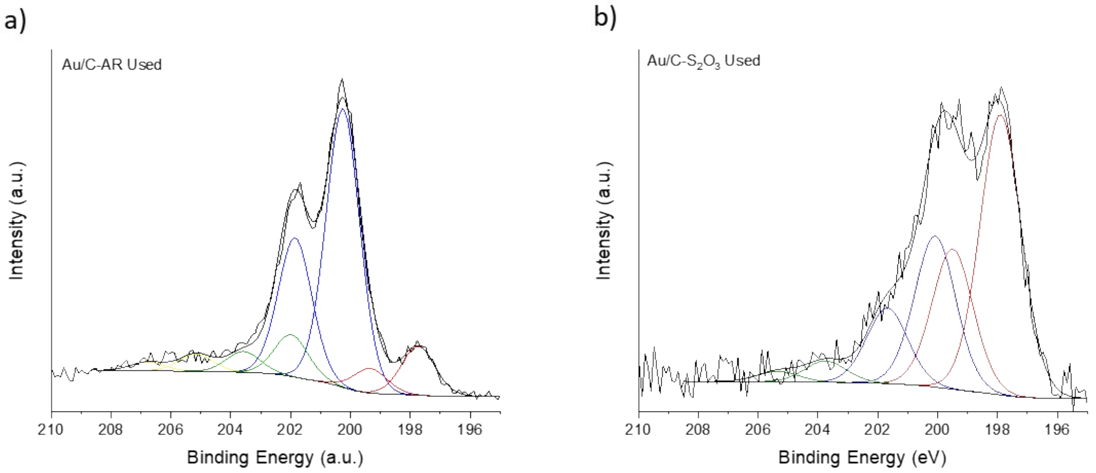

Figure S11. S K-edge a) normalised absorption and b) normalised first derivative of standards and samples acquired *ex situ*.

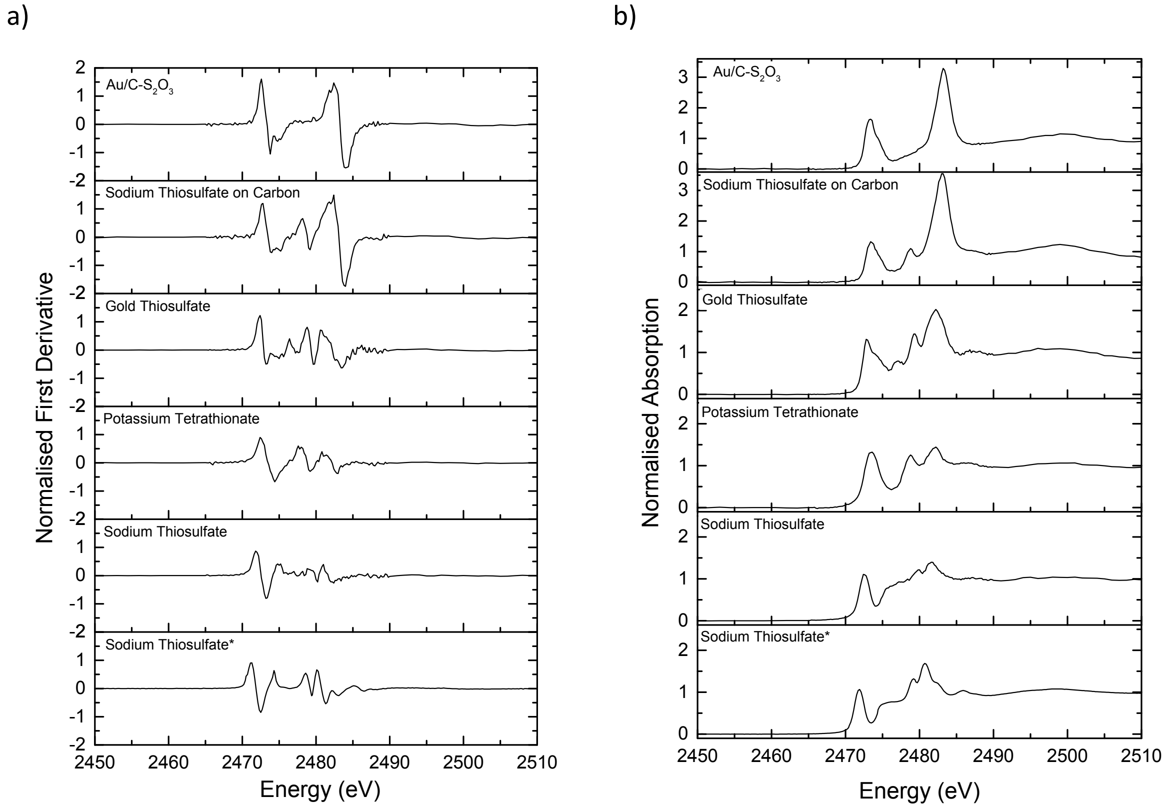

(Sodium thiosulfate on carbon has been prepared *via* a "washing step" which reproduced the conventional catalyst preparation method without using the metal precursor. In particular, *ca.* 2 g of carbon, previously grounded, were mixed with *ca.* 5mL of an aqueous solution containing $Na_2S_2O_3$).

Figure S12. a) 2D and b) 3D plot of all *in situ* S K edge XANES normalised absorption spectra of the $Au/C\text{-}S_2O_3$ catalyst acquired while heating to reaction temperature (200 °C).

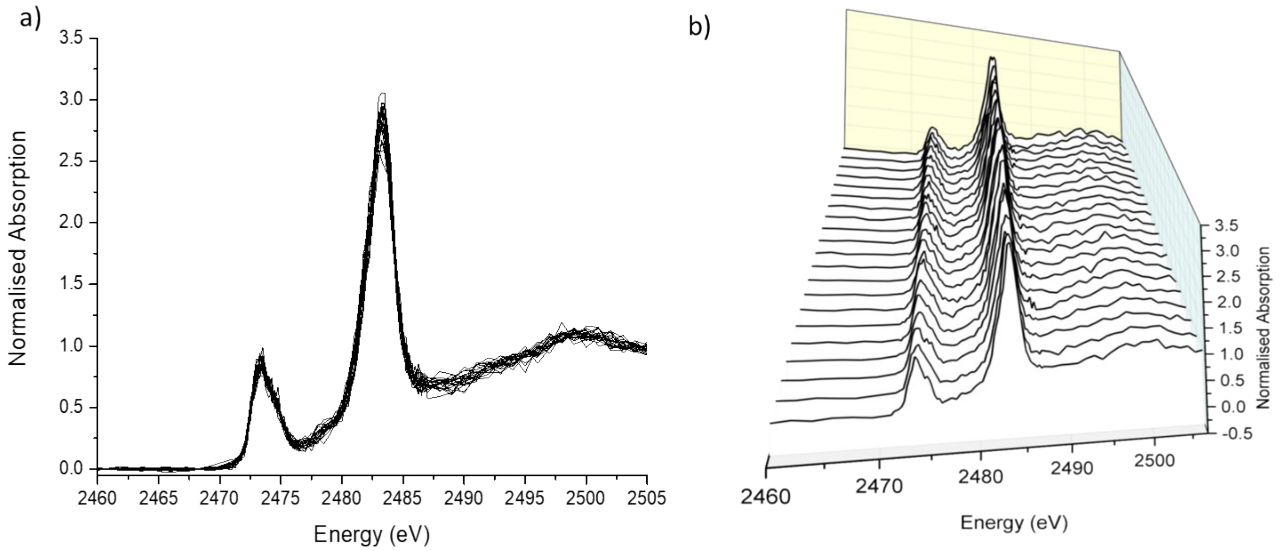

Figure S13. a) 2D and b) 3D plot of all *in situ* S K-edge normalised XANES spectra of the $Au/C\text{-}S_2O_3$ catalyst during the time-on-line.

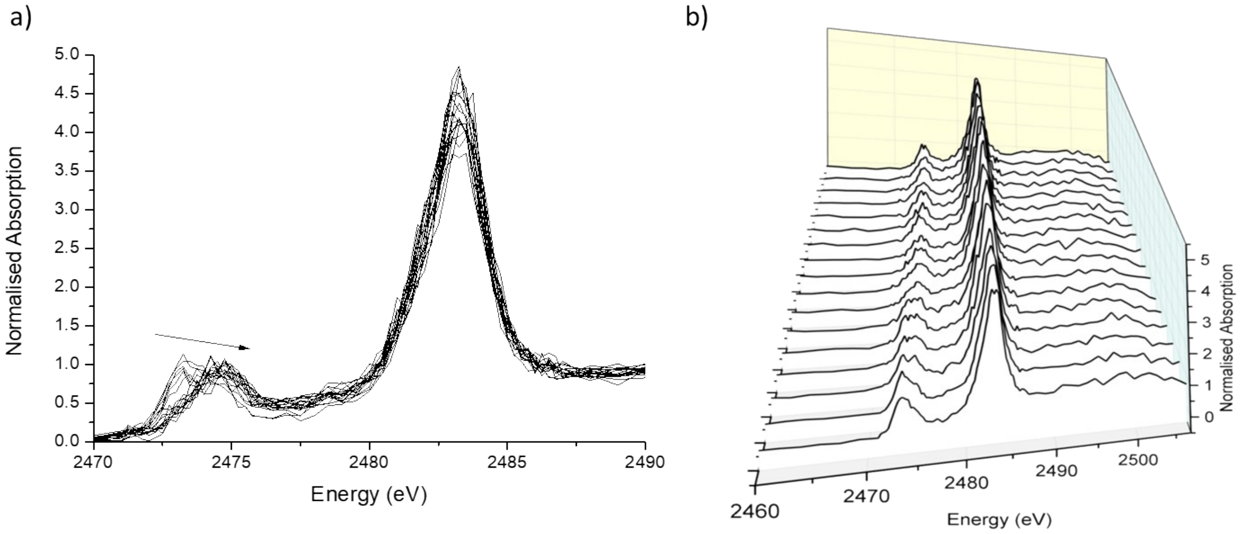

Figure S14. *Ex situ* S 2p XPS of the a) fresh and b) used Au/C-S_2O_3 catalysts.

See table S4 for quantification of the S content.

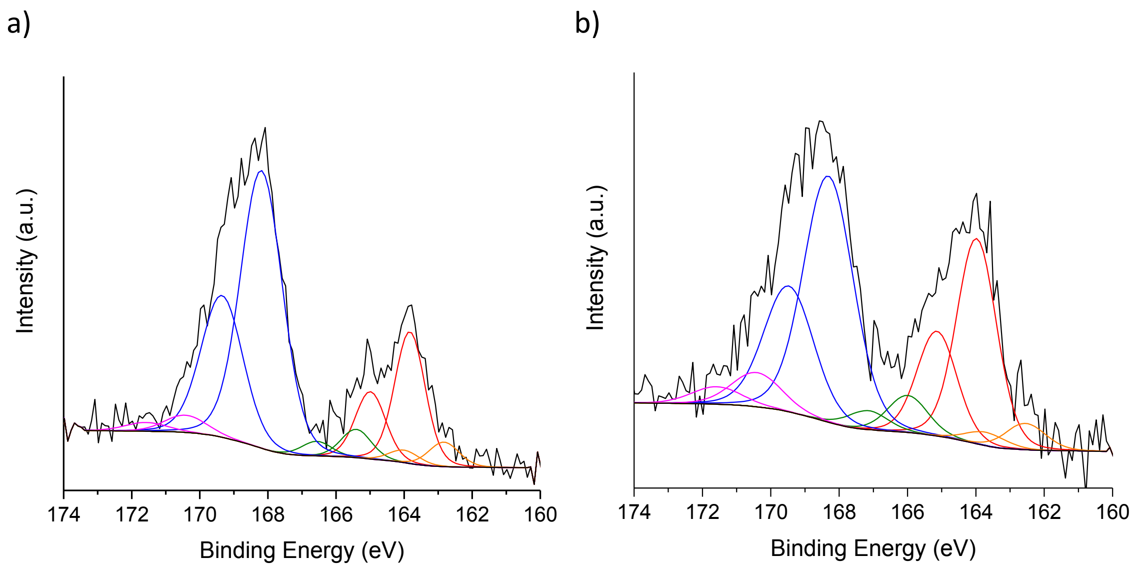

Figure S15. VCM productivity as function of time-on-line during the "accelerated deactivation test" for the Au/C-S_2O_3 catalyst.

(i) $HCl/C_2H_2/Ar$, (ii) HCl/Ar, (iii) second $HCl/C_2H_2/Ar$, (iv) C_2H_2/Ar, and (v) third $HCl/C_2H_2/Ar$ exposure.

Test conditions: 90 mg catalyst, 23.5 mL min^{-1} C_2H_2, 23.7 mL min^{-1} HCl and 2.7 mL min^{-1} Ar, 200 °C.

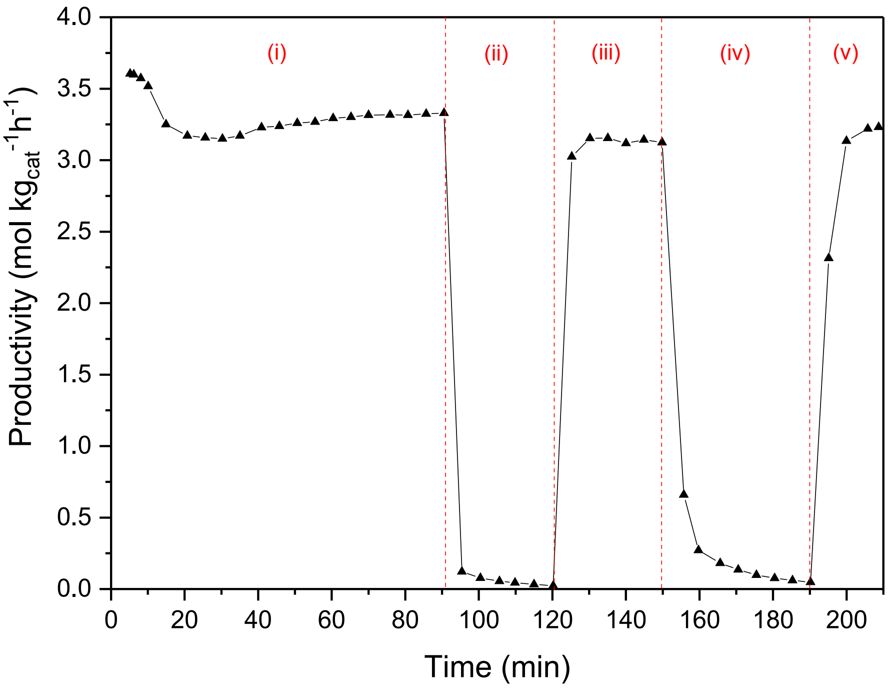

Figure S16. Comparison of the k space for the measured Au L_3-edge during step (i) $HCl/C_2H_2/Ar$ and step (iv) C_2H_2/Ar of catalyst treatment reported in figure S15.

Test conditions: 90 mg catalyst, 23.5 mL min^{-1} C_2H_2, 23.7 mL min^{-1} HCl and 2.7 mL min^{-1} Ar, 200 °C.

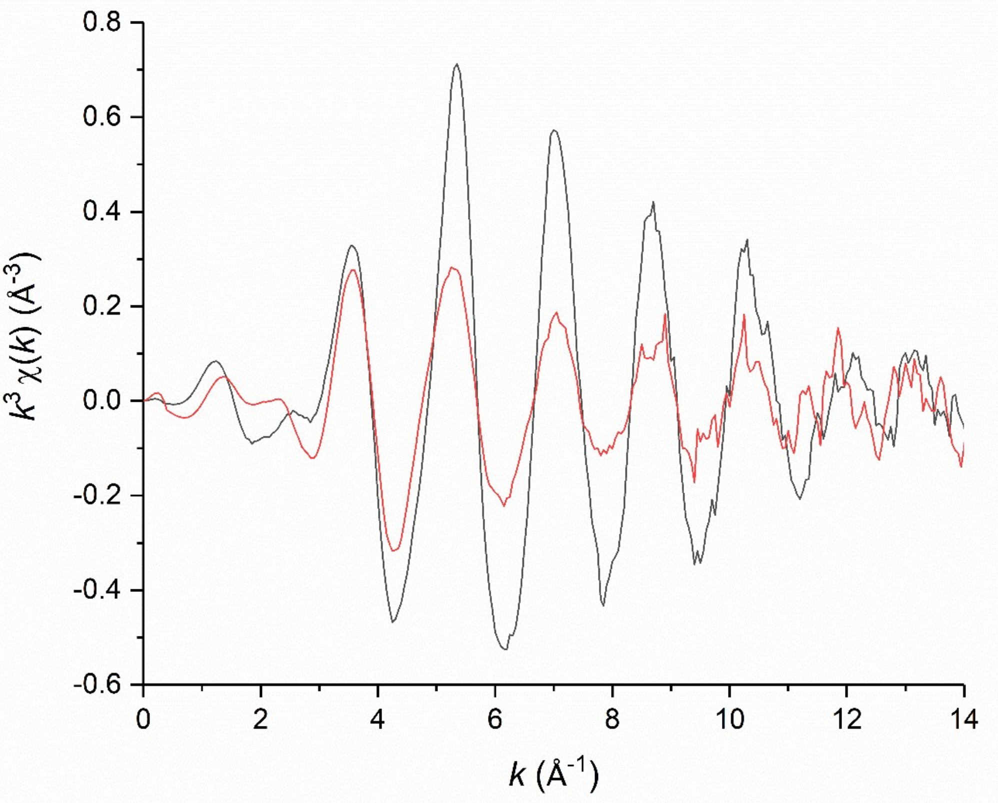

Figure S17. Comparison of k space for the measured Au L_3-edge and fitting model using combined Au-C and Au-Cl paths during step (iv) C_2H_2/Ar of catalyst treatment reported in figure S15.

Test conditions: 90 mg catalyst, 23.5 mL min^{-1} C_2H_2, 23.7 mL min^{-1} HCl and 2.7 mL min^{-1} Ar, 200 °C.

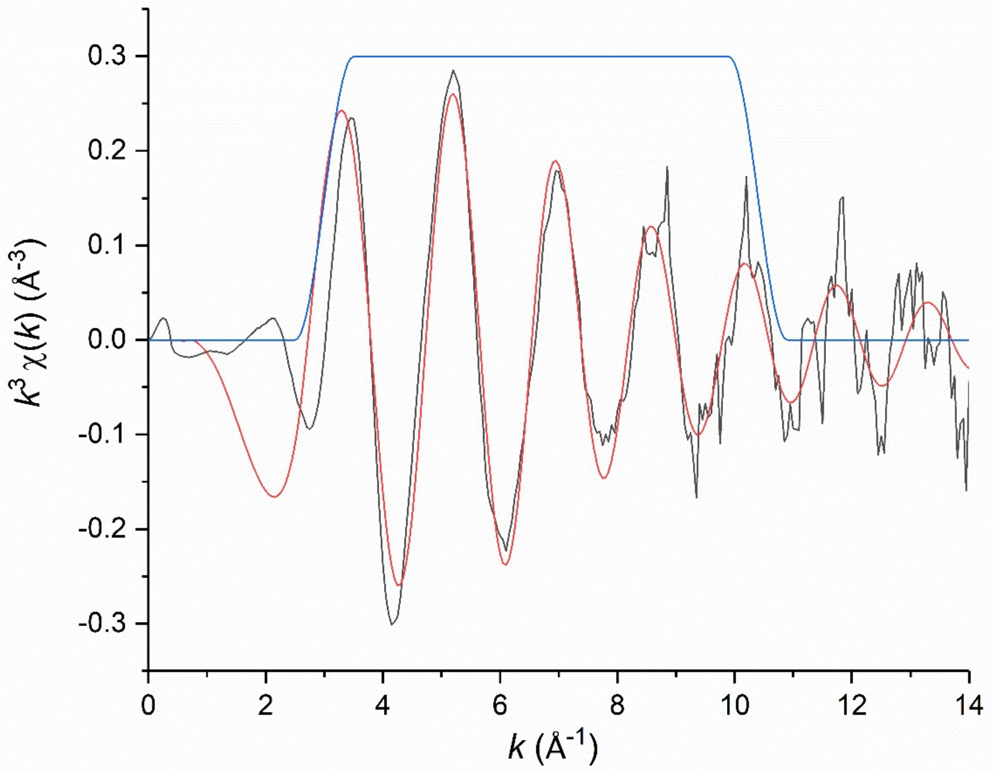

References

1. V.A. Solé, E. Papillon, M. Cotte, Ph. Walter, J. Susini, *Spectrochim. Acta Part B*, 2007, **62**, 63-68.

2. a) 18. S.E. Shadle, B. Hedman, K. O. Hodgson and E. I. Solomon, *J. Am. Chem. Soc.,* 1995, **117**, 2259- 2212. b) T. Glaser, B. Hedman, K. O. Hodgson and E. I. Solomon, *Acc. Chem. Res.,* 2000, **33**, 859-868.

3. M. Newville, *Rev. Mineral. Geochem*. 2014, **78**, 33–74.

4. B. Ravel and M. Newville, *J. Synchrotron Radiat.,* 2005, **12**, 537–541.
